# Potential Inhibitors of Galactofuranosyltransferase 2 (GlfT2): Molecular Docking, 3D-QSAR, and *In Silico* ADMETox Studies

**DOI:** 10.1038/s41598-019-52764-8

**Published:** 2019-11-19

**Authors:** Christopher Llynard D. Ortiz, Gladys C. Completo, Ruel C. Nacario, Ricky B. Nellas

**Affiliations:** 1 Institute of Chemistry, College of Arts and Sciences, University of the Philippines Los Banos, College, Laguna, 4031 Philippines; 2 Institute of Chemistry, College of Science, University of the Philippines Diliman, Diliman, Quezon City, 1101 Philippines

**Keywords:** Computational biology and bioinformatics, Drug discovery, Diseases, Chemistry

## Abstract

A strategy in the discovery of anti-tuberculosis (anti-TB) drug involves targeting the enzymes involved in the biosynthesis of *Mycobacterium tuberculosis*’ (*Mtb*) cell wall. One of these enzymes is Galactofuranosyltransferase 2 (GlfT2) that catalyzes the elongation of the galactan chain of *Mtb* cell wall. Studies targeting GlfT2 have so far produced compounds showing minimal inhibitory activity. With the current challenge of designing potential GlfT2 inhibitors with high inhibition activity, computational methods such as molecular docking, receptor-ligand mapping, molecular dynamics, and Three-Dimensional-Quantitative Structure-Activity Relationship (3D-QSAR) were utilized to deduce the interactions of the reported compounds with the target enzyme and enabling the design of more potent GlfT2 inhibitors. Molecular docking studies showed that the synthesized compounds have binding energy values between −3.00 to −6.00 kcal mol^−1^. Two compounds, #27 and #31, have registered binding energy values of −8.32 ± 0.01, and −8.08 ± 0.01 kcal mol^−1^, respectively. These compounds were synthesized as UDP-Galactopyranose mutase (UGM) inhibitors and could possibly inhibit GlfT2. Interestingly, the analogs of the known disaccharide substrate, compounds #1–4, have binding energy range of −10.00 to −19.00 kcal mol^−1^. The synthesized and newly designed compounds were subjected to 3D-QSAR to further design compounds with effective interaction within the active site. Results showed improved binding energy from −6.00 to −8.00 kcal mol^−1^. A significant increase on the binding affinity was observed when modifying the aglycon part instead of the sugar moiety. Furthermore, these top hit compounds were subjected to *in silico* ADMETox evaluation. Compounds #31, #70, #71, #72, and #73 were found to pass the ADME evaluation and throughout the screening, only compound #31 passed the predicted toxicity evaluation. This work could pave the way in the design and synthesis of GlfT2 inhibitors through computer-aided drug design and can be used as an initial approach in identifying potential novel GlfT2 inhibitors with promising activity and low toxicity.

## Introduction

Tuberculosis (TB) has been one of the fatal diseases worldwide^1–3^. The causative agent of this disease is a pathogenic microorganism, *Mycobacterium tuberculosis* (*Mtb*), which is a rod-shaped mycobacteria^4^. The World Health Organization (WHO) recently reported that one-third of world’s population^5^ has TB. There is still a slow decline on the number of infected individuals for the previous years^6^, concluding that current efforts to fight this disease is challenging. In the Philippines, TB has been identified as the sixth leading cause of morbidity and mortality, which makes the country ranked sixth out of the top 22 TB burdened countries worldwide^7^. In addition, the Philippines is considered one of the countries with the highest number of cases of multi-drug resistant tuberculosis (MDR-TB)^8^. Despite active efforts in administering the latest medical treatment to prevent the spread of TB, this pulmonary illness remains a global threat.

There are two kinds of resistance to TB: (a) mono and (b) poly resistant. The latter is divided into two: (a) multi-drug resistant (MDR-TB), to at least two of the standard or first-line anti-TB drugs, and (b) extensively/extreme-drug resistant (XDR-TB), to at least two of the first-line anti-TB drugs and is immune to the second-line anti-TB drugs. The inconsistent TB treatment for patients has led to a new strain that is totally drug-resistant (TDR-TB) to either the first-line or second-line anti-TB drugs^9,10^. There is currently no known treatment for TDR-TB; thus, this opens up the challenge of developing new strategies on design of new anti-TB drugs.

*Mycobacterium* species’ cell wall acts as a rigid scaffold that hinders the penetrating action of antibiotics. Some commercially-available anti-TB drugs (i.e. ethambutol or isoniazid) are used to disrupt the cell wall biosynthesis^11–13^, in combination with other anti-TB drug (i.e. rifampicin or streptomycin) that have intracellular targets^14^. Current anti-TB treatment, i.e. DOTS^15^, targets the *Mtb* cell wall’s integrity and allow the facile permeation of the antibiotics to the organism. The microorganism’s ability to adapt and develop resistance to these drugs is still a challenge. This continuing battle is evident in the emergence of resistant strains responsible for MDR-, XDR-, and TDR-TB in some regions of the world. Hence, a potential strategy for TB treatment is in the search for novel compounds that can interfere with the *Mtb*’s cell wall complex biosynthesis.

*Mtb*’s cell wall efficiently protects the mycobacteria from detrimental factors during the infection stage, at the same time, it could also be the weak spot of the organism^16^. One component of the *Mtb*’s cell wall is the arabinogalactan (AG) complex. This is a unique structure that is composed of D-galactofuranosyl and L-arabinofuranosyl monosaccharides^17^. The AG complex, like any other carbohydrate polymers, is essential for mycobacterial viability^18,19^. Thus, the enzymes involved in its biosynthesis might serve as putative therapeutic targets. One of the enzymes involved in the synthesis of this complex is Galactofuranosyltransferase 2 (GlfT2), an enzyme that catalyzes the transfer of galactofuranosyl residues from UDP-Gal*f* to the growing galactan chain^20,21^. This chain is composed of ~30 D-galactofuranose (Gal*f*) residues that are linked via alternating *β*-(1 → 5) and *β*-(1 → 6) linkages with the reducing end covalently attached to a linker disaccharide consisting of rhamnose and N-acetylglucosamine^22^.

Several active researches have zoomed in on GlfT2 inhibitors as anti-TB drugs. Current strategies in the design of GlfT2 inhibitors involved mimicking either the donor or acceptor substrate of the enzyme^16,21,23^. Uridine diphosphate-galactofuranose (UDP-Gal*f*) is considered the donor substrate of GlfT2. The approach used is to design a glycomimetic compound with disrupted hydrogen-bonding interaction with the enzyme’s catalytic site, specifically with D372, through modification of the 6-OH position^16^. With this strategy, four compounds were synthesized such as the UDP-6-fluoro-*α*-D-Gal*f* (compound #49), the UDP-*β*-L-Ara*f* (compound #50), the UDP-5-deoxy-*α*-D-Gal*f* (compound #51), and the UDP-6-deoxy-*α*-D-Gal*f* (compound #52). Compounds #50 and #52 were found to inhibit the production of the glycolipids and compounds #49 and #51 have effectively reduced the length of galactolipid produced^16^.

The drawback of using the aforementioned compounds is in their inability to pass through the cell membrane because of the polar sugar moiety and charged diphosphate group. Thus, another strategy was proposed by replacing the diphosphate group of UDP-Gal*f* with basic amino acids such as lysine, glutamine, tryptophan, and histidine. Also, instead of using D-Gal*f*, L-Ara*f* was used. Among the four sugar-amino acid-nucleosides, those with tryptophan and histidine as the replacement for the diphosphate moiety have shown 30% and 37% inhibition activity with GlfT2^23^, respectively.

**Figure 1:**
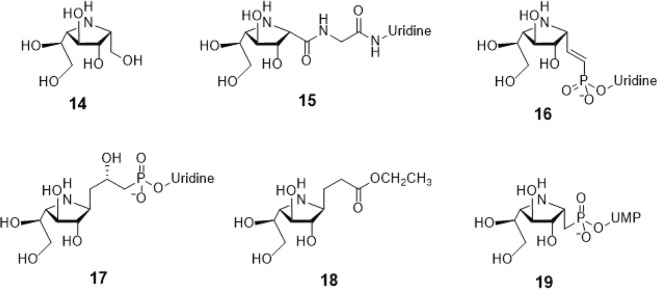
Structures of Imino-Sugars as GlfT2 inhibitors.

Another series of GlfT2’s donor-mimi*cking inhibitors are compo*unds #15–18 (Fig. 1). These are structurally described as imino-galactofuranose sugar moieties with uridine as an aglycon. These compounds only differ in linker composition that is an amide (compound #15)^24^, phosphate and double bond (compound #16)^25,26^ and phosphate and a hydroxyl group (compound #17)^27^. Compound #18 (Fig. 1) is considered an analog of compound #17^28^. Furthermore, compound #19 (Fig. 4) is an imino-galactofuranose with UDP as an aglycon. These probes were previously used as UDP-galactopyranose mutase (UGM) inhibitors^26^. It was found that only compound #18 exhibited less than 35% inhibition activity against UGM^26^.

**Figure 2:**
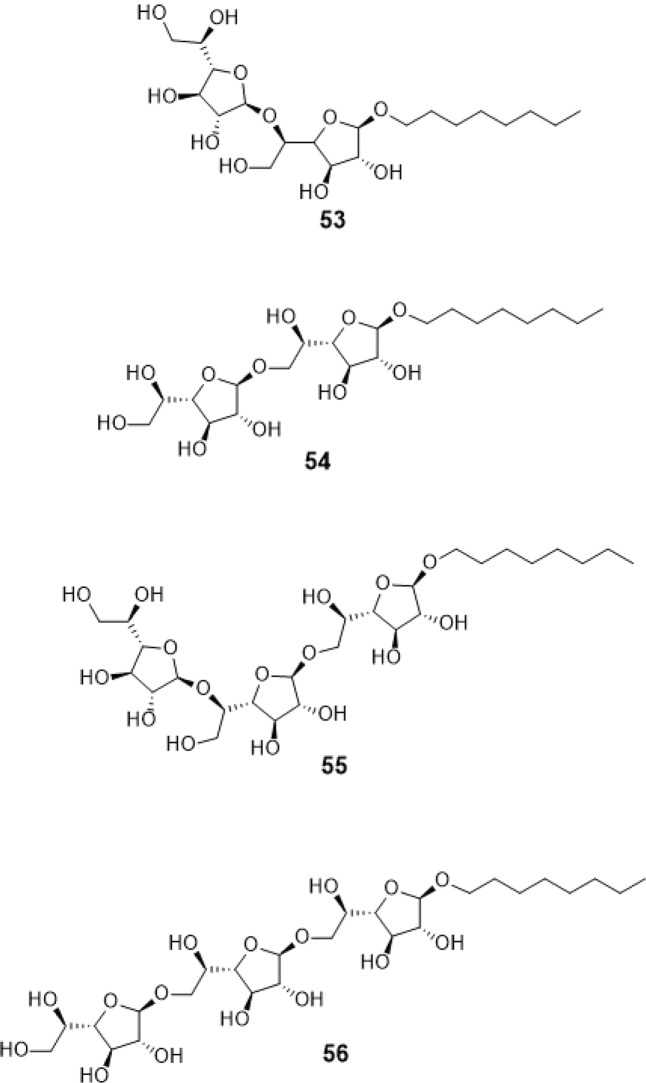
Structures of Synthetic Acceptor Substrates as GlfT2 inhibitors.

A previous study synthesized compounds #53–56 (Fig. 2) to evaluate GlfT’s specificity^16^. These compounds are octyl di- and tri-saccharides. Using kinetic characterization of GlfT with these compounds, it was found that trisaccharides were better substrates than disaccharides. The current strategy in designing for the acceptor mimic substrates were based from these results.

Aside from mimicking the donor and acceptor substrates to inhibit GlfT2, making a resemblance of the positive character of the transition state of the substrate was also used. A recent study synthesized 14 sulfonium ions with varying side chains. These are expected to mimic the transition state during the glycosyl transfer reaction. Among these compounds, the one having a sulfonium ion with 12-hydroxydodecyl side chain exhibited the highest inhibition activity of 60%^29^.

**Figure 3:**
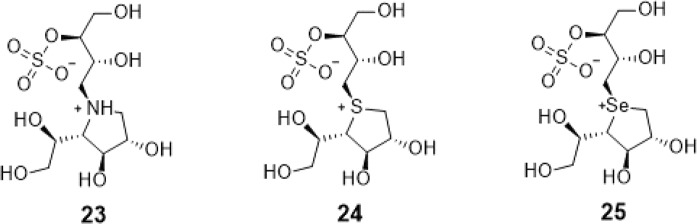
Structures of the Transition State Mimics as GlfT2 inhibitors.

Both glycosidase and galactofuranosyltransferase carry out glycosyl transfer reaction, hence, it is noteworthy that the proposed inhibitors of glycosidase may also potentially serve as GlfT2 inhibitors^29^. Compound #24 (Fig. 3) is one of the two naturally-occurring sulfonium ion glycosidase inhibitors, known as salacinol^30^. This compound was found to have an inhibitory activity towards *α*-glycosidase. Compounds #23 and #25 (Fig. 3) are both salacinol-derivatives replacing sulfur atom with nitrogen and selenium, respectively^31,32^.

With regard to the charged-sulfur containing compounds as GlfT2 inhibitors, a series of galactofuranosyl N,N’-dialkyl sulfenamides and sulfonamide were synthesized. Here, it was found that compounds with shorter N,N’-dihexyl chains showed low inhibition activity compared with compounds having longer dioctyl and didecyl chains^34^.

The potential GlfT2 inhibitors aforementioned have low inhibition activities with the enzyme. As such, it is timely to shift and find another strategy on designing new GlfT2 inhibitors. *In silico* studies such as molecular docking and 3D-QSAR are now possible with the availability of the GlfT2 crystal structure (PDB ID: 4FIX)^20^. Molecular dynamics (MD) simulations were performed to obtain ensemble of protein structures to be used for molecular docking studies. Also, 3D-QSAR was used as guide in designing new GlfT2 inhibitors. Moreover, the top hit compounds were screened using *in silico* ADMETox.

## Results and Discussion

### Ensemble docking

The 100 different protein conformations were obtained from the entire 100 ns simulation using the clustering analysis of the cpptraj^35^ module of the AMBERTools 15 package^36^. Previously synthesized and functionalized compounds were prepared using the MarvinSketch software^37^. Subsequently, molecular docking was performed using AutoDock Vina^38–40^. Here, the inhibition constant (K*i*) was obtained from the binding energy (ΔG) using the formula: K*i* = exp(ΔG/RT), where R is the universal gas constant (1.985 × 10^−3^ kcal mol^−1^ K^−1^) and T is the temperature (298.15 K). The previously synthesized and functionalized compounds were docked to obtain the binding energy of the complexes formed between the receptor and the ligands. The natural acceptor substrate was found to have a binding energy of −6.63 ± 0.02 kcal mol^−1^ and Y236, D256, W309, K369, D372, W399, and Q409 as key interacting amino acids with the natural substrate. The binding energy of the natural donor substrate will be used as the reference value here.

### First line Anti-TB drug

**Figure 4:**
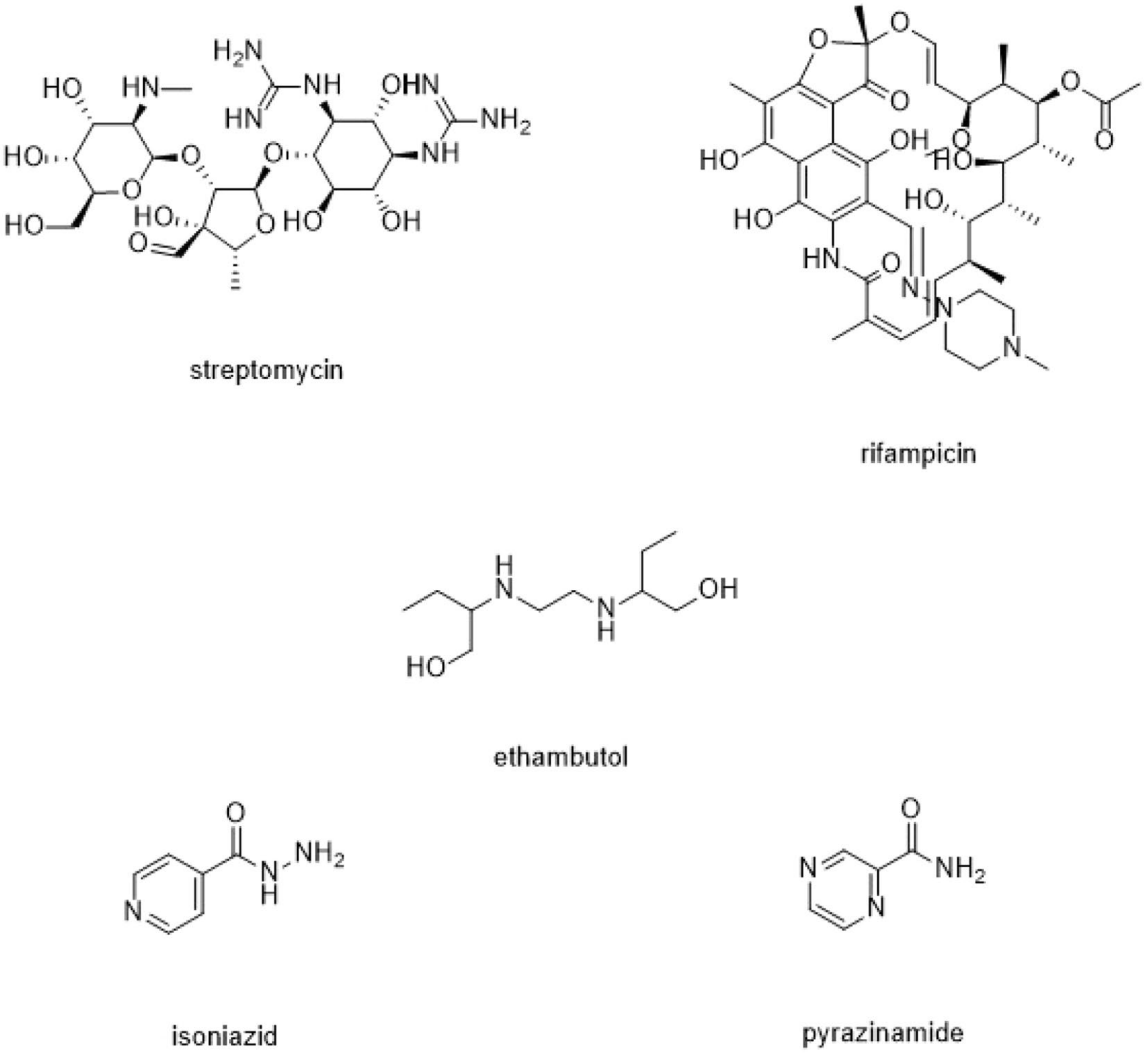
Structures of the first-line Anti-TB drugs.

Several anti-TB drugs developed were categorized according to target. One classification is the first-line anti-TB drugs (Fig. 4) which inhibits the synthesis of the bacterial cell wall essential for its pathogenicity^41^ and virulence^42^. The first-line anti-TB drug compounds were subjected to molecular docking studies. Results showed that the binding energy values indicate that these compounds weakly bind to the active site. Majority of the first-line anti-TB drugs such as ethambutol, isoniazid and pyrazinamide were found to have no interaction with the key GlfT2 active site amino acids (Table 2). Overall, the observed low binding energies of these compounds originated from the weak to lack of interaction with the amino acids within the active site (Table 1).

**Table 1:** Binding affinities and Inhibition constant (T = 298.15 K) of first-line Anti-TB drugs.

Compounds	Binding energy (kcal/mol)	Inhibition constant, µM
**Natural substrate**	−6.63 ± 0.02	14
**streptomycin**	−6.25 ± 0.01	26
**rifampicin**	−6.19 ± 0.04	29
**ethambutol**	−4.12 ± 0.01	950
**isoniazid**	−4.44 ± 0.01	553
**pyrazinamide**	−4.04 ± 0.01	1078

**Table 2:** Interacting amino acids with First-line anti-TB drugs.

Compounds	Major interacting amino acids								Additional interacting amino acids						
**Natural substrate**	Y236	D256	W309	W348	K369	D372	W399	Q409							
**Streptomycin**	Y236			W348	K369				P167		G232	H396	I368	F169	
**Rifampicin**					K369										
**Ethambutol**															
**Isoniazid**										Q200					
**Pyrazinamide**									P167		G232		I368		T168

### Current GlfT2 inhibitors in the literature

Recently, one of the strategies used to synthesized GlfT2 inhibitors is by mimicking the identified donor, acceptor, and transition-state substrates^43–45^.

#### Mimicking GlfT2’s donor substrate as GlfT2 inhibitor

**Figure 5:**
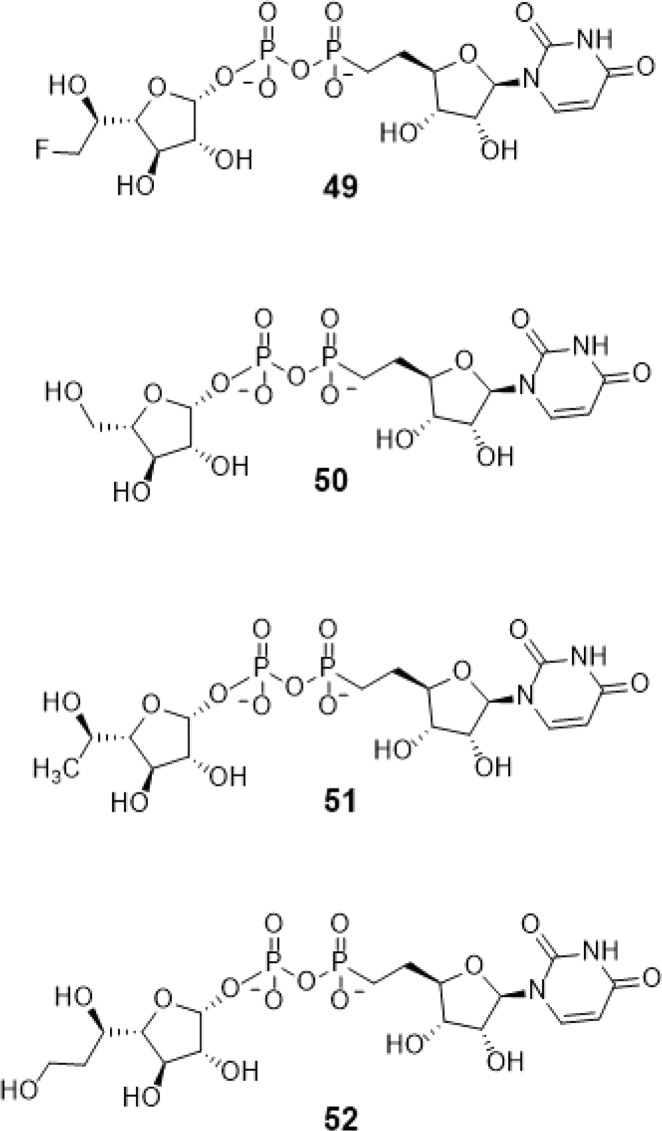
Structures of UDP-furanoses as GlfT2 inhibitors.

**Figure 6:**
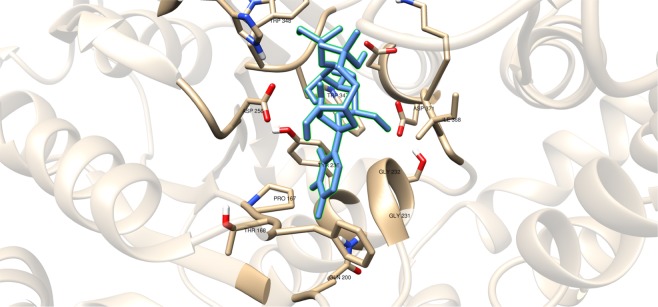
Three-dimensional plot of the interaction of compound #49 with GlfT2’s active site.

Compounds having a similar structure with the GlfT2’s donor substrate could be used as potential GlfT2 inhibitors. In a previous study, donor substrate mimics such as compounds #49–52 (Fig. 5) were examined using a spectrophotometric assay with GlfT1 and GlfT2. It was found that these compounds inhibit GlfT2 except for compound #50^16^. Docking results showed that the binding energy values of compounds #50 and #52 (Fig. 5) were relatively lower than the reference value (Table 11). On the other hand, with respect to the reference value, compounds #49 and #51 (Fig. 5), exhibited lower binding energy values of −6.79 ± 0.03 and −6.72 ± 0.10 kcal mol^−1^, respectively (Table 11). Furthermore, it showed that compounds #49, #51, and #52 interact with Y236, W348, P167, G232, and D371 (Fig. 6 and Table 12). Intriguingly, compound #50 has lost its hydrophobic interaction with D256, I368, and T168. In contrary, these amino acids were found to interact with compounds #49, #51 and #52 (Table 12). The absence of these amino acids interacting with compound #50 could be the reason for its weak binding affinity with GlfT2 as described previously^16^.

**Figure 7:**
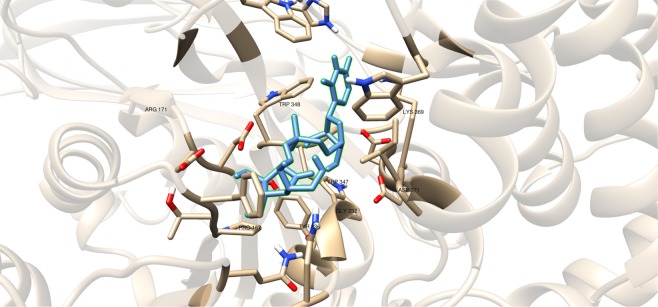
Three-dimensional plot of the interaction of compound 16 with GlfT2’s active site.

As discussed previously, compounds #15–19 were found to be UGM inhibitors and GlfT2 donor-substrate mimics (Fig. 4). Thus, all these compounds were subjected to molecular docking studies to probe whether the UGM inhibitors could also act as GlfT2 inhibitors. Results showed that the binding energy values of compounds #15, #16 and #19 were relatively higher than the reference value (Table 3). Compound #17 was found to have a lower binding energy value of −6.36 ± 0.01 kcal mol^−1^ compared with the reference value. Compound #18, compound #17’s analog, was found to have a binding energy value of −5.02 ± 0.01 kcal mol^−1^, relatively much lower compared with compound #17. The absence of a UDP moiety in compound #18, which was present in compound #17, could be the possible reason for its low binding affinity. The linker seems to increase the interaction of the ligand with the amino acid residues within the active site through hydrogen bonding. The interactions among the linker’s phosphate group, Y236, and G232 were also observed with compounds #15–17 and #19. The presence of this kind of interaction could be the reason for the stabilization of these compounds within the active site (Fig. 7 and Table 4).

**Table 3:** Binding affinities and Inhibition constant (T = 298.15 K) of Imino-Sugars as GlfT2 inhibitors.

Compounds	Binding energy (kcal/mol)	Inhibition constant, µM
**Natural substrate**	−6.63 ± 0.02	14
**14**	−4.18 ± 0.01	856
**15**	−6.69 ± 0.01	12
**16**	−6.83 ± 0.01	10
**17**	−6.36 ± 0.01	21
**18**	−5.02 ± 0.01	209
**19**	−6.72 ± 0.01	12

**Table 4:** Interacting amino acids with Imino-Sugars as GlfT2 inhibitors.

Compounds	Major interacting amino acids								Additional interacting amino acids									
**Natural substrate**	Y236	D256	W309	W348	K369	D372	W399	Q409										
**14**	Y236	D256		W348					P167	R171		W347						
**15**	Y236	D256		W348					P167	R171	G232	W347	I368					
**16**	Y236			W348	K369	D372			P167	R171	G232			F169	D371			
**17**	Y236	D256			K369	D372			P167		G232	W347	I368	F169	D371	T168	G231	
**18**	Y236	D256		W348	K369	D372			P167		G232	W347			D371			
**19**	Y236	D256		W348	K369	D372			P167	R171	G232	W347	I368	F169	D371		G231	Q200

#### Mimicking GlfT2’s acceptor substrate as GlfT2 inhibitor

**Figure 8:**
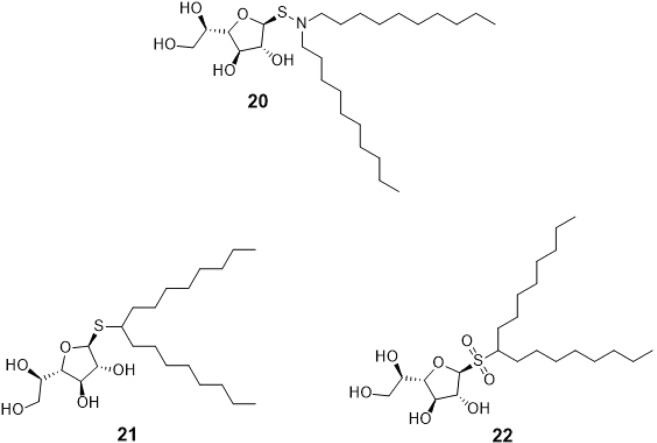
Structures of Sulfenamide and Sulfonamides as GlfT2 inhibitors.

Compounds #20 (galactofuranosyl N,N’-didecyl sulfenamide), #21 (galactofuranosyl dioctyl thioglycoside), and #22 (sulfone derivative of compound #21 via the oxidation of sulfur) (Fig. 8) were screened for inhibition effect using disk susceptibility test assay^46^. Results revealed that compound #20 exhibited an inhibitory effect comparable to the shorter diakyl chains. On the other hand, compounds #21 and #22 were found to have an inhibitory effect with GlfT2 at a concentration less than 5 *μ*M^46^.

**Figure 9:**
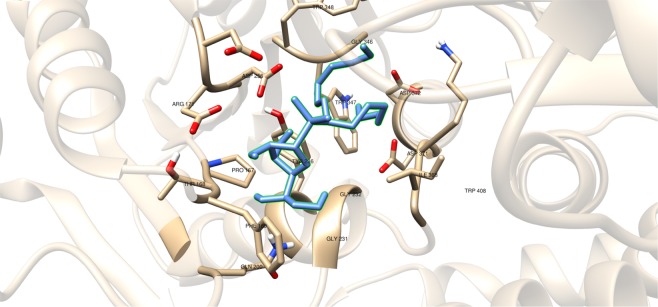
Three-dimensional plot of the interaction of compound #22 with GlfT2’s active site.

These compounds were subjected to molecular docking studies and showed lower binding energy values compared with the reference value (Table 5). Among these three, compound #22 (Fig. 8) has highest binding energy due to the additional oxygen (sulfone) that was observed to interact with D371 and D372 (Fig. 9). The dialkyls were observed to have hydrophobic interactions with R171, Y236, D371, D372, G232, W347, W348, and D256 (Table 6).

**Table 5:** Binding affinities and Inhibition constant (T = 298.15 K) of Sulfenamide and Sulfonamides as GlfT2 inhibitors.

Compounds	Binding energy (kcal/mol)	Inhibition constant, µM
**Natural substrate**	−6.63 ± 0.02	14
**20**	−5.80 ± 0.01	55
**21**	−5.80 ± 0.01	55
**22**	−5.98 ± 0.01	41

**Table 6:** Interacting amino acids with Sulfenamide and Sulfonamides as GlfT2 inhibitors.

Compounds	Major interacting amino acids								Additional interacting amino acids											
**Natural substrate**	Y236	D256	W309	W348	K369	D372	W399	Q409												
**20**	Y236	D256		W348	K369	D372			P167		G232	W347		F169	D371	T168				
**21**	Y236	D256				D372			P167	R171	G232	W347		F169	D371		G231	Q200		
**22**	Y236	D256		W348		D372			P167	R171	G232	W347	I368	F169	D371	T168	G231	Q200	G346	W408

**Figure 10:**
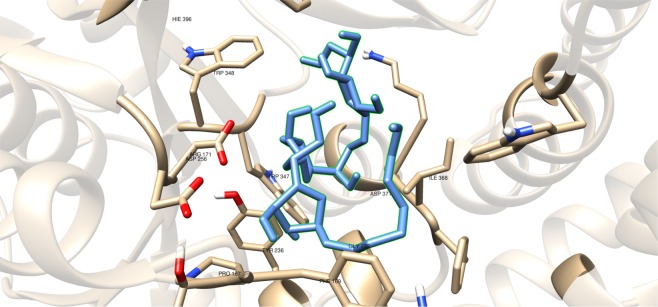
Three-dimensional plot of the interaction of compound #55 with GlfT2’s active site.

Compounds #53–56 (Fig. 2) were synthesized to evaluate the specificity of GlfT^16^. Using kinetic characterization of GlfT with these compounds, it was found that trisaccharides were better substrates than disaccharides^21^. Docking studies were performed on these compounds and the results showed that trisaccharides, compounds #55 and #56, have lower binding energy values (−6.24 ± 0.04 and −6.19 ± 0.04 kcal mol^−1^, respectively) compared with disaccharides, compounds #53 and #54 (−6.16 ± 0.03 and −6.11 ± 0.03 kcal mol^−1^, respectively) (Table 13). The additional sugar moiety of a trisaccharide compared to a disaccharide extended the long alkyl chain, allowing interaction with W408 via hydrophobic interaction (Fig. 10 and Table 14). This could be the origin of the enhanced interaction observed in both the experimental and docking studies.

#### UDP-Galactopyranose mutase (UGM) substrate as GlfT2 inhibitor

Another strategy used to inhibit GlfT2 was by repurposing compounds that were proposed to be inhibitors of enzymes involved in the galactan chain synthesis. One of these enzymes is UDP-Galactopyranose mutase (UGM). In the absence of galactofuranose in mammals^33^, UGM catalyzes the conversion of UDP-galactopyranose to UDP-galactofuranose which will eventually be the donor substrate of GlfT2^26,47^. Here, some of the proposed UGM inhibitors were subjected to molecular docking studies with GflT2.

Compound #14 (Fig. 1) was reported to be a weak inhibitor of UDP-Gal*p* mutase^47^. Upon molecular docking investigation the binding energy was found to be much lower, −4.18 ± 0.01 kcal mol^−1^, compared with the reference value (Table 3). Furthermore, among the key GlfT2 active site amino acids that were previously discussed, Y236, D256 and W348, were the only amino acids observed to be interacting with compound #14. The decrease in the number of interacting amino acids with compound #14 is one of the reasons for its poor binding affinity.

A recent study have discussed the development of microtiter plate-based assay to screen uridine-based compounds against UGM^48^. Among the compounds in the uridine-based library used in the assay, only compound #27 (Fig. 12) was found to be a weak inhibitor (IC_50_ = 6.0 *μ*M). Its binding energy value (−8.32 ± 0.01 kcal mol^−1^) was observed to be lower than the reference value (Table 9). The interactions of the compound with P167, G232, W347, I368, D371, T168, W408, M285 and H296 may account for its improved binding affinity (Fig. 13).

**Figure 11:**
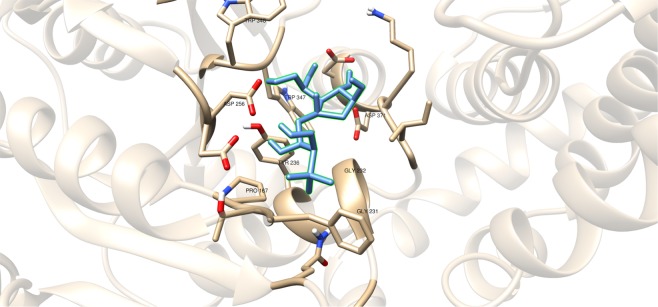
Three-dimensional plot of the interaction of compound #25 with GlfT2’s active site.

**Figure 12:**
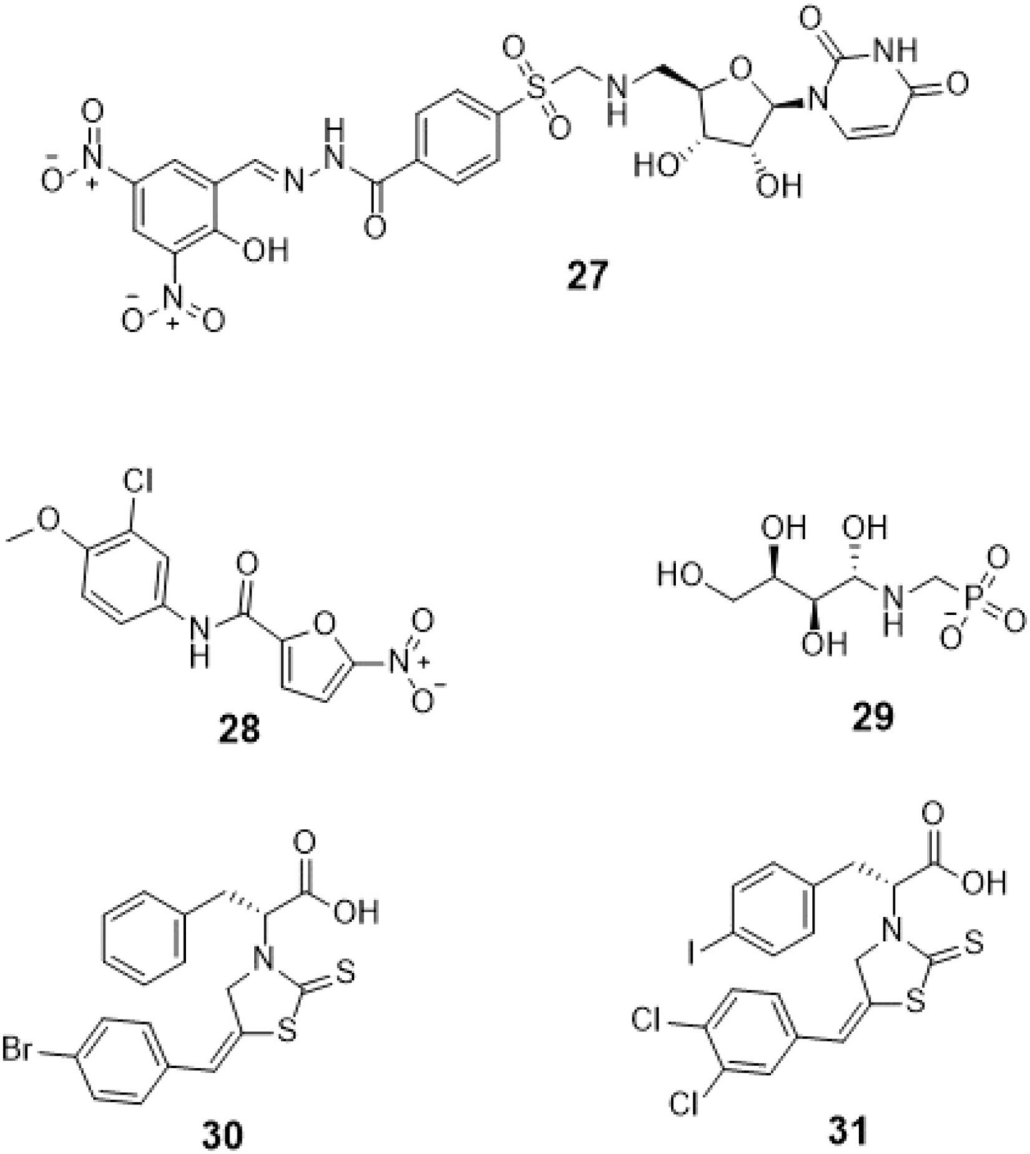
Structures of UDP-Galactopyranose mutase (UGM) inhibitors as GlfT2 inhibitors.

**Table 7:** Binding affinities and Inhibition constant (T = 298.15 K) of Transition State Mimics as GlfT2 inhibitors.

Compounds	Binding energy (kcal/mol)	Inhibition constant, µM
**Natural substrate**	−6.63 ± 0.02	14
**23**	−4.99 ± 0.01	217
**24**	−5.31 ± 0.01	127
**25**	−5.60 ± 0.01	78

**Table 8:** Interacting amino acids with Transition State Mimics as GlfT2 inhibitors.

Com-pounds	Major interacting amino acids								Additional interacting amino acids											
**Natural substrate**	Y236	D256	W309	W348	K369	D372	W399	Q409												
**23**	Y236	D256		W348		D372			P167	R171	G232	W347	I368	F169	D371	T168				
**24**	Y236	D256				D372			P167	R171	G232	W347		F169	D371		G231	Q200		
**25**	Y236	D256		W348		D372			P167	R171	G232	W347	I368	F169	D371	T168	G231	Q200	G346	W408

**Table 9:** Binding affinities and Inhibition constant (T = 298.15 K) of Synthesized Halogenated GlfT2 inhibitors.

Compounds	Binding energy (kcal/mol)	Inhibition constant, µM
**Natural substrate**	−6.63 ± 0.02	14
**27**	−8.32 ± 0.01	1
**28**	−7.13 ± 0.01	6
**29**	−3.96 ± 0.01	1242
**30**	−7.79 ± 0.01	2
**31**	−8.08 ± 0.01	1

**Figure 13:**
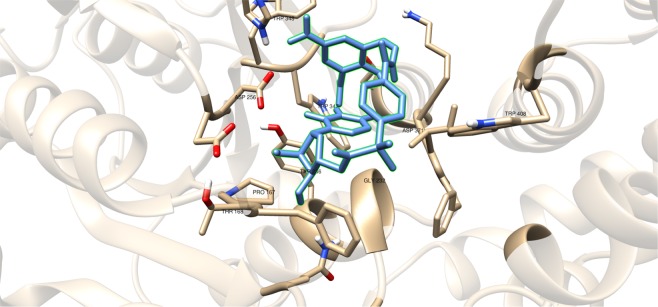
Three-dimensional plot of the interaction of compound #27 with GlfT2’s active site.

Aside from being a known antibacterial agent for urinary tract infection treatment, compound #28 (Fig. 12) was further used as a UGM inhibitor and found to moderately inhibit the enzyme^49^. Docking this with GlfT2 resulted in a lower binding energy compared with the reference value (Table 9). The absence of the interaction of this compound with W347, I368 and T168 may account for this lower binding affinity compared with compound #27.

Compounds #30 and #31 showed a promising activity towards UGM (IC_50_ = 1.6 *μ*M)^50,51^. These compounds differ in the type and number of halides they contain. Compound #30 has only bromide and compound #31 has two chloride and one iodide atom present in the 5-arylidene-2-thioxo-4-thiazolidinone (ATT) core. Results of the molecular docking studies showed that compound #31 have a lower binding energy compared with compound #30 (Table 9). The observed interaction of Q200 with compound #30’s chloride atom and K369 with iodide atom may account for the difference in the binding energy values between the two compounds (Table 10).

**Table 10:** Interacting amino acids with synthesized halogenated compounds as GlfT2 inhibitors.

Com-pounds	Major interacting amino acids								Additional interacting amino acids														
**Natural substrate**	Y236	D256	W309	W348	K369	D372	W399	Q409															
**27**	Y236	D256		W348					P167		G232	W347	I368		D371	T168				W408	M285	H396	
**28**	Y236					D372			P167	R171	G232			F169	D371		G231						
**29**	Y236			W348	K369	D372					G232	W347			D371								
**30**	Y236								P167		G232		I368	F169		T168			N229				F367
**31**	Y236			W348					P167		G232	W347	I368				G231	Q200

**Table 11:** Binding affinities and Inhibition constant (T = 298.15 K) of Synthetic UDP-furanoses.

Compounds	Binding energy (kcal/mol)	Inhibition constant, µM
**Natural substrate**	−6.63 ± 0.02	14
**49**	−6.79 ± 0.03	10
**50**	−6.57 ± 0.03	15
**51**	−6.72 ± 0.10	12
**52**	−6.42 ± 0.03	19

Compound #29 (Fig. 12) showed no inhibition or poor inhibitory activity with UGM^51^. Molecular docking studies have found that it only registered a binding energy of −3.96 ± 0.01 kcal mol^−1^ which was much lower compared with the reference value. The derivative of the sugar moiety may have occupied only the sugar binding region of the active site and do not interact with the residues within the UDP binding region. This suggests that the residues interacting with UDP contribute to the binding of UDP-Gal*f*, and the absence of UDP or replacement of any moiety that could mimic it, may account for the compound’s low binding affinity.

Among the reported synthesized GlfT2 inhibitors that were included in this study, compounds #27 and #31 (Fig. 12) were found to have binding energies of −8.32 ± 0.01 kcal mol^−1^ and −8.08 ± 0.01 kcal mol^−1^, respectively (Tables 9 and 10). The observed higher binding energy values compared with GlfT2’s natural substrate seems to originate from the interaction of W347, W348, and W408 (only for compound #27) with the aromatic rings present in the compound. The presence of tryptophan within the active site could stabilize the inhibitors via *π*-*π* interaction with the aromatic rings.

#### Mimicking GlfT2’s transition-state substrate as GlfT2 inhibitor

Aside from mimicking the GlfT2 donor and acceptor substrates, mimicking the GlfT2 transition-state substrate (positively charged moiety) was another interesting strategy. Since *α*-glycosidase and galactofuranosyltransferase carry out glycosyl transfer reaction, it was proposed that *α*-glycosidase inhibitors could also be potential GflT2 inhibitors^29^. With this, molecular docking studies using compounds #23−25^31^ (Fig. 3) generate binding energies of −4.99 ± 0.01, −5.31 ± 0.01, and −5.60 ± 0.01 kcal mol^−1^, respectively (Table 7).

The positively charged moiety of these compounds were found to interact with D371 and the other interacting amino acids as shown in Fig. 11 and Table 8. Still, lower binding energy values were observed compared with the reference value (Table 7). The structural motif and binding energy values of compounds #23–25 were comparable with compound #18. These compounds only have a sugar moiety and a short linker (Figs. 1 and 3). As previously discussed, the absence of a moiety that could interact with UDP binding region amino acids could account for the low binding affinity of a molecule within GlfT2’s active site.

#### Newly Designed Sugar Furanosides as GlfT2 Inhibitors

*In silico* drug design draws attention among researches because it is time-saving and cheap. Computer-aided drug design uses computational tools to discover, develop, and analyze drugs^52^. One technique used for drug design is ligand-based computer-aided drug design which involves ligands that are known to interact with target receptor, and account for the binding strength of a given molecule by knowing the nature of the interactions^53^.

Functionalizing a drug with an azide group has been largely used in the pharmaceutical industries^54,55^. It was then recognized as a novel pharmacophore in medicinal chemistry especially in the emergence of zidovudine, an anti-retroviral drug for the treatment of Acquired Immuno-Deficiency Syndrome (AIDS). Also, azide group was used in tumor-labelling^56^ for cancer treatment. It is noteworthy that azido-substituted drugs have high affinity towards the target receptor^57^. It was found that tetrahydroimidazobenzodiazepinthiones (TIBO) or thiourea derivatives are potential drugs for treating TB^58,59^. Guanidine derivative drugs also pose a promising role in medicinal chemistry because of their anticancer^60,61^, antiviral^62^, antibacterial^63^ properties. Streptomycin, a known anti-TB drug, has two guanidino groups. The aforementioned functional groups such as azido, thiourea and guanidino groups were used here in designing new GlfT2 inhibitors because of their antibacterial property and high receptor affinity.

**Figure 14:**
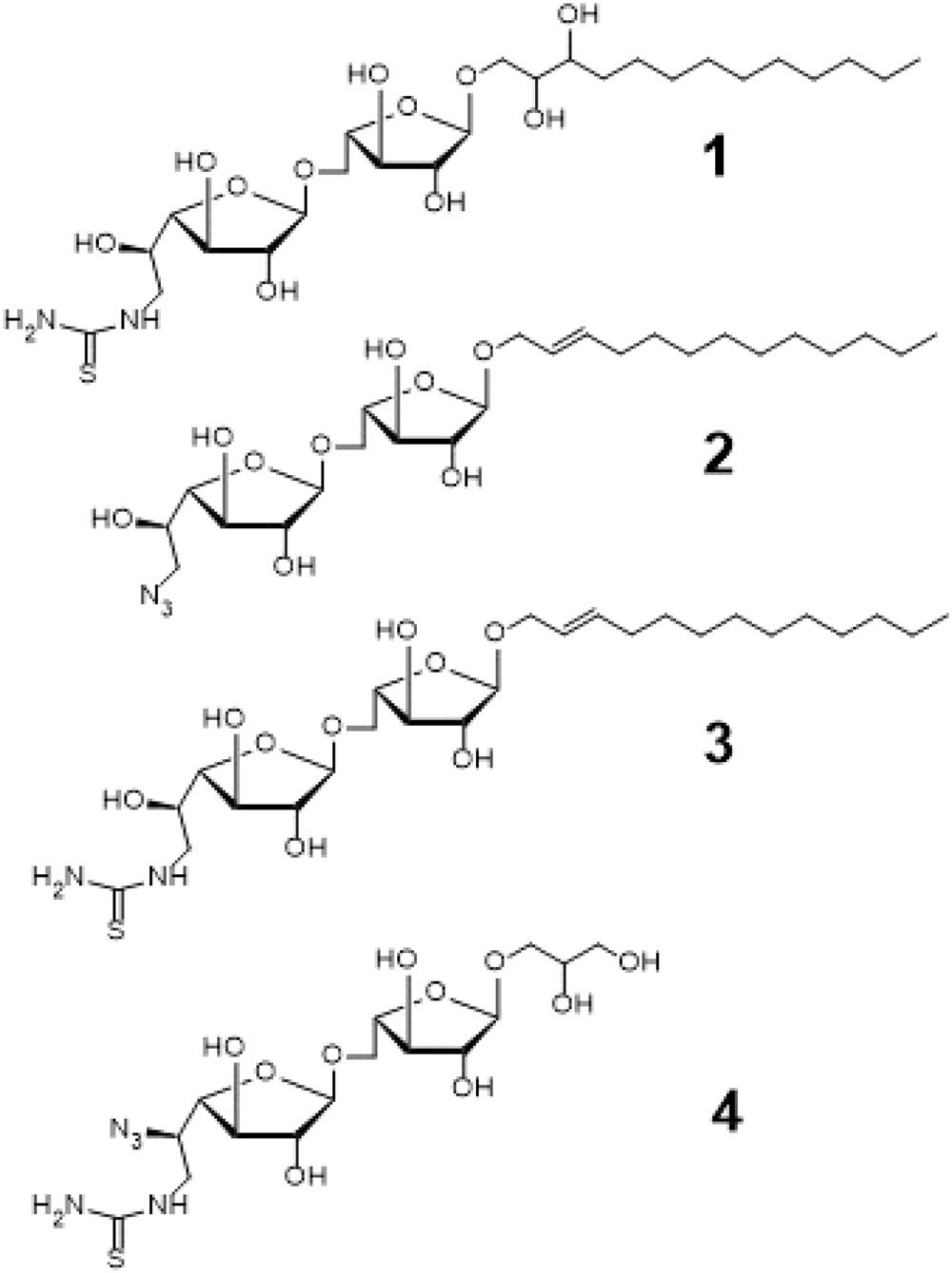
Structures of Newly Designed Acceptor Substrates as GlfT2 inhibitors.

The design was an analog of the known GlfT2 substrate dissacharide, octyl *β*-D-galactofuranosyl-(1→5)- *β*-L-arabino-furanoside. They found that substrates with longer chain aglycon were better substrates of glycosyltransferases in *Mycobacterium* species^64^. Compounds #2 and #3 (Fig. 14) were both trans-2-tridecen-1-yl glycosides having a modification in the non-reducing end wherein the 6-OH position was replaced with azido and thiourea functional groups, respectively. On the other hand, compound #1 was obtained via the oxidation of double bond in the aglycon of compound #3. It was found that compound #3 has a higher binding energy value of −10.32 ± 0.02 kcal mol^−1^ compared with compound #2 with binding energy value of −11.08 ± 0.02 kcal mol^−1^. It is proposed that the presence of azido group have a higher binding affinity compared with the thiourea group which is evident on the binding energies presented.

 From the binding energies of compounds #1 and #3, it can be observed that compound #1 has a lower binding energy of −14.67 ± 0.04 kcal mol^−1^ compared with compound #3 having −10.32 ± 0.02 kcal mol^−1^ (Table 15). There were observed interactions among D256,Y236, and thiourea and another set of interactions among D371, D372, the hydroxyl groups of the disaccharide, and the aglycon of compound #1 (Table 16). The addition of two hydroxyl groups on the aglycon effectively increased the enzyme-substrate interaction within the active site.

**Table 12:** Interacting amino acids with Synthetic UDP-furanoses as GlfT2 inhibitors.

Compounds	Major interacting amino acids								Additional interacting amino acids							
**Natural substrate**	Y236	D256	W309	W348	K369	D372	W399	Q409								
**49**	Y236	D256		W348					P167		G232	W347	I368	F169	D371	T168
**50**	Y236			W348		D372			P167	R171	G232				D371	
**51**	Y236	D256		W348	K369	D372			P167		G232	W347	I368		D371	T168
**52**	Y236	D256		W348	K369	D372			P167		G232	W347	I368		D371	T168

**Table 13:** Binding affinities and Inhibition constant (T = 298.15 K) of Synthetic Acceptor Substrates.

Compounds	Binding energy (kcal/mol)	Inhibition constant, µM
**Natural substrate**	−6.63 ± 0.02	14
**53**	−6.16 ± 0.03	30
**54**	−6.11 ± 0.03	33
**55**	−6.24 ± 0.04	26
**56**	−6.19 ± 0.04	29

**Table 14:** Interacting amino acids with Synthetic Acceptor Substrates as GlfT2 inhibitors.

Com-pounds	Major interacting amino acids								Additional interacting amino acids												
**Natural substrate**	Y236	D256	W309	W348	K369	D372	W399	Q409													
**53**	Y236	D256		W348		D372			P167		G232	W347	I368	F169	D371		G231	Q200			N229
**54**	Y236	D256			K369	D372			P167		G232	W347	I368	F169	D371	T168	G231			H396	
**55**	Y236	D256		W348	K369				P167	R171	G232	W347	I368	F169	D371				W408		
**56**	Y236	D256		W348	K369	D372			P167		G232	W347	I368				G231		W408

**Table 15:** Binding affinities and Inhibition constant (T = 298.15 K) of newly designed GlfT2 inhibitors.

Compounds	Binding energy (kcal/mol)	Inhibition constant, µM
**Natural substrate**	−6.63 ± 0.02	14
**1**	−14.67 ± 0.04	1.71 × 10−4
**2**	−11.08 ± 0.02	1.95 × 10−3
**3**	−10.32 ± 0.02	2.7 × 10−1
**4**	−19.23 ± 0.05	7.7 × 10−9

**Table 16:** Interacting amino acids with Newly Designed Acceptor Substrates as GlfT2 inhibitors.

Com-pounds	Major interacting amino acids								Additional interacting amino acids													
**Natural substrate**	Y236	D256	W309	W348	K369	D372	W399	Q409														
**1**	Y236	D256		W348		D372			P167		G232	W347	I368	F169	D371		G231	Q200				
**2**	Y236	D256		W348	K369	D372			P167		G232	W347	I368	F169	D371	T168	G231					H396
**3**	Y236	D256		W348	K369	D372			P167	R171	G232	W347	I368	F169	D371					W408		
**4**	Y236	D256		W348	K369	D372			P167		G232	W347	I368				G231		D258	W408	Y344	

**Figure 15:**
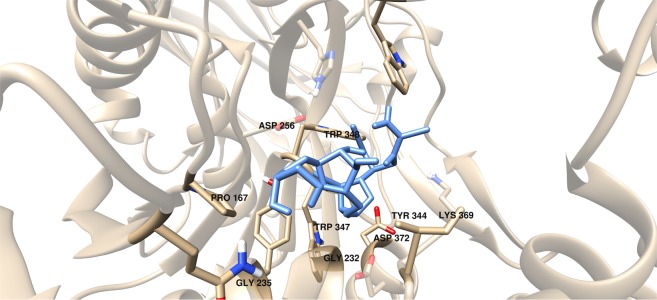
Three-dimensional plot of the interaction of compound #4 with GlfT2’s active site.

Compound #4 (Fig. 14), a glyceryl glycoside, has a modification in the non-reducing end wherein the 5-OH and 6-OH position were replaced with an azido and guanidino functional groups, respectively. As observed, D256 and I368 interacts with the hydroxyl groups of glyceryl aglycon (Table 16). Y344, P167 and D258 were observed to interact with the guanidino group and D372 was observed to interact with the azide group (Fig. 15 and Table 16). These residues interact with the inhibitor through hydrogen bonding and seem to be the origin for the observed high binding affinity. Among the newly designed sugar-based inhibitors, compound #4 is the most promising compound with a binding energy of −19.23 ± 0.05 kcal mol^−1^.

#### Redesigned Sugar Furanosides as GlfT2 Inhibitors using 3D-QSAR

Structures of the synthesized GlfT2 inhibitors were subjected to 3D-QSAR to improve the structural motif of the inhibitors. The 3D-QSAR is an important tool on providing substantial information about the molecular attribute essential for biological activity of compounds^65,66^.

Results showed that the Pearson coefficient is R^2^ = 0.99 which signifies the reliability of the test and training sets used. The structures of the presented synthesized GlfT2 inhibitors were aligned along their respective molecular field points. Figure 20 shows negative steric field points (green field) which indicate that steric groups should be avoided on that particular part of the molecule. Whereas, positive steric field points (yellow field) indicate that steric groups should be added on that particular part of the molecule. Moreover, negative electrostatic field points indicate that an electrostatic contributor i.e. negatively charged group/hydrogen-bond acceptor, should be added on that particular part of the molecule. Whereas, positive electrostatic field points indicate that an electrostatic contributor i.e. positively charged group/hydrogen-bond donor, should be added on that particular part of the molecule.

From this, insights on designing the top 18 hit compounds were acquired. From 3D-QSAR, instead of having a flexible long chain aglycon, the aglycon was replaced with cholesterol (compound #58), tocopherol (compound #62), retinol (compound #61) cholesterol derivatives i.e. calciferol, calcitriol (compound #63), cholecalciferol (compounds #59, #60 and #64) and cholecalciferol derivatives (compounds #66–75) which are more rigid and sterically hindered.

**Figure 16:**
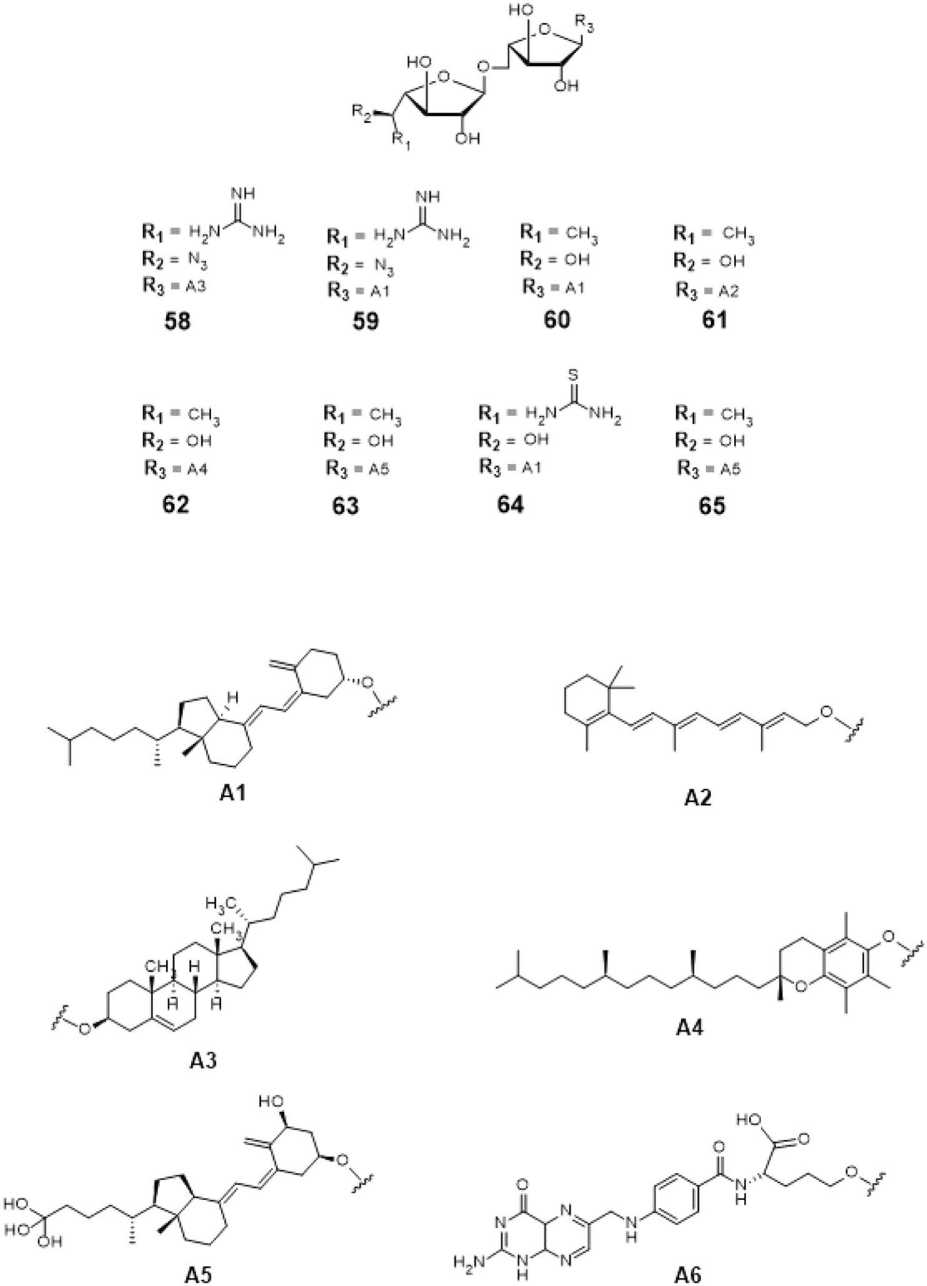
Structures of the 3D-QSAR designed compounds.

**Figure 17:**
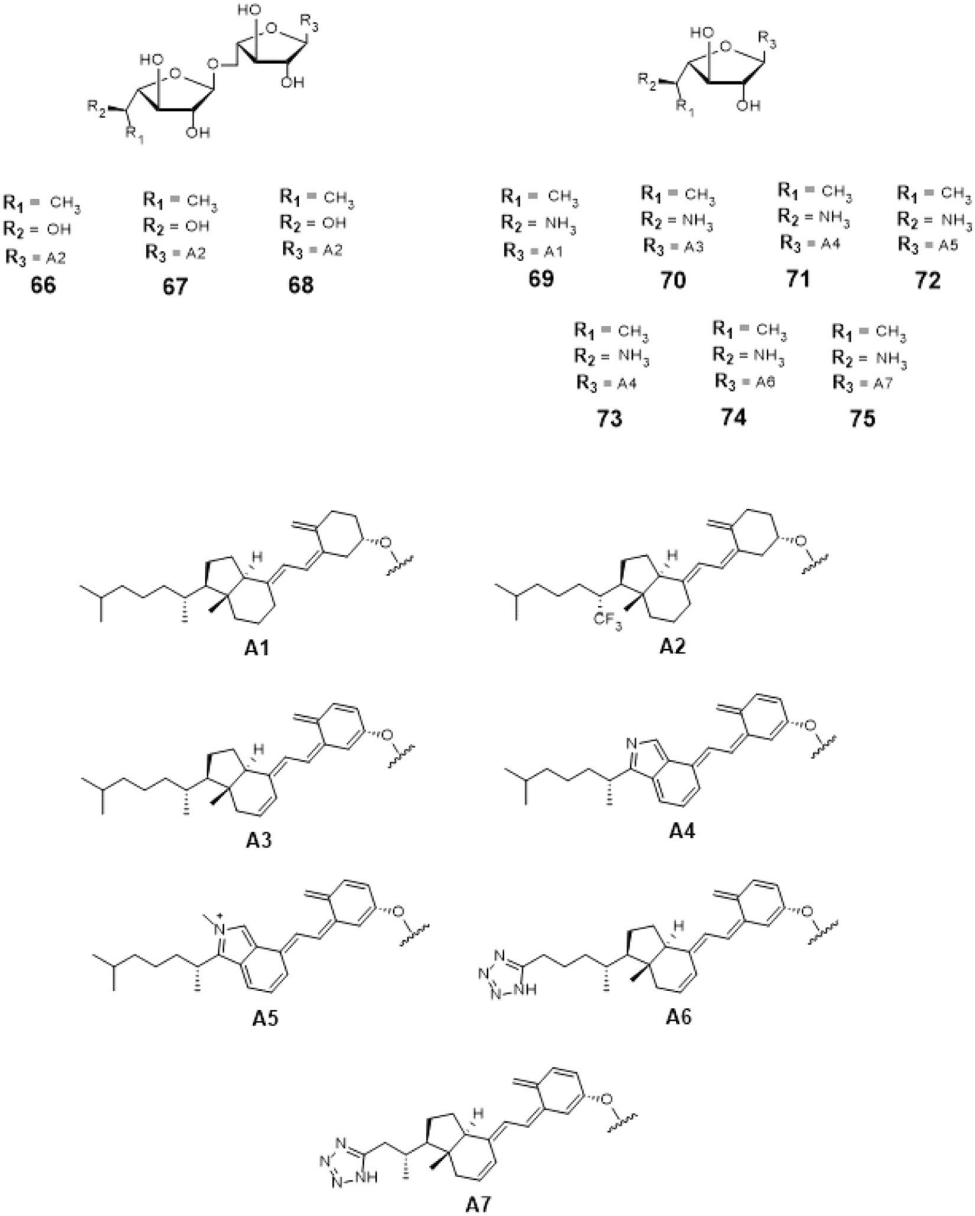
3D-QSAR Designed Possible Inhibitors.

The 5-OH and 6-OH position of the non-reducing end of the substrates were functionalized with azido- and guanidino-group, respectively for compounds #58 and #59 (Figs. 16 and 17). For compounds #66–75 (Figs. 16 and 17), the 5-OH and 6-OH position of the non-reducing end of the substrates were functionalized with amine- and methyl group. For compounds #60–63 and #65 (Figs. 16 and 17), only 6-OH position of the non-reducing end of the substrates were functionalized with a methyl group. Lastly, for compound #64 (Fig. 16), the 6-OH position of its non-reducing end was functionalized with a thiourea group.

These 18 newly 3D-QSAR designed structures, compounds #58–75 (Figs. 16 and 17) were subjected to ensemble docking to reassess the binding affinity of the compounds. The compounds registered binding energy values of ~−8.00 kcal mol^−1^ from having a binding energy values of ~−6.00 kcal mol^−1^ (Tables 17 and 19).

**Table 17:** Binding affinities and Inhibition constant (T = 298.15 K) of 3D-QSAR designed compounds.

Compounds	Binding energy (kcal/mol)	Inhibition constant, µM
**Natural substrate**	−6.63 ± 0.02	14
**58**	−8.28 ± 0.05	1
**59**	−8.29 ± 0.05	1
**60**	−8.35 ± 0.05	1
**61**	−8.24 ± 0.05	1
**62**	−8.10 ± 0.06	1
**63**	−8.21 ± 0.05	1
**64**	−8.08 ± 0.04	1
**65**	−7.64 ± 0.05	2

**Table 18:** Interacting amino acids with 3D-QSAR designed compounds.

Compounds	Major interacting amino acids								Additional interacting amino acids														
**Natural substrate**	Y236	D256	W309	W348	K369	D372	W399	Q409															
**58**		D256	W348	K369					G232	W347	I368	F169			G231		H396						
**59**	Y236	D256	W348	K369	D372	Q409	P167	R171	G232	W347	I368	F169				Q200				K402	Y348		
**60**	Y236	D256			D372				G232	W347	I368	F169	D371	T168		Q200	H396					Y344	W309
**61**	Y236		W348	K369	D372					W347								E300					
**62**	Y236		W348	K369	D372	Q409	P167			W347	I368		D371					E300					
**63**	Y236	D256	W348	K369	D372		P167	R171	G232	W347	I368	F169	D371	T168	G231	Q200	H396		M286	K402			
**64**	Y236	D256	W348	K369				R171				F169	D371	T168		Q200	H396	E300	M286	K402			
**65**	Y236	D256	W348	K369			P167		G232	W347			D371						M286	K402

**Table 19:** Binding affinities and Inhibition constant (T = 298.15 K) of 3D-QSAR designed compounds.

Compounds	Binding energy (kcal/mol)	Inhibition constant, µM
**Natural substrate**	−6.63 ± 0.02	14
**66**	−8.47 ± 0.05	1
**67**	−8.36 ± 0.06	1
**68**	−8.41 ± 0.04	1
**69**	−8.23 ± 0.05	1
**70**	−8.51 ± 0.05	1
**71**	−8.21 ± 0.05	1
**72**	−8.28 ± 0.05	1
**73**	−8.17 ± 0.05	1
**74**	−8.69 ± 0.05	1
**75**	−8.67 ± 0.05	1

Results show that the modification of the aglycon instead of the sugar moiety lead to the significant increase on the binding energy of designed compounds.

**Figure 18:**
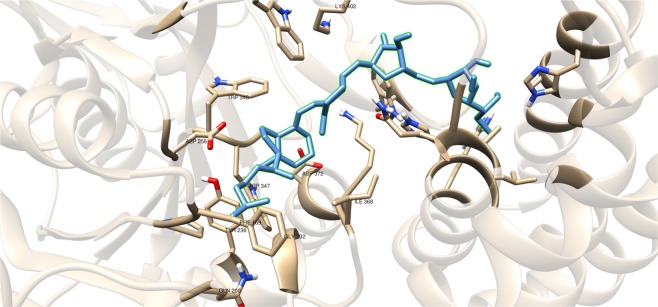
Three-dimensional plot of the interaction of compound #60 with GlfT2’s active site.

**Figure 19:**
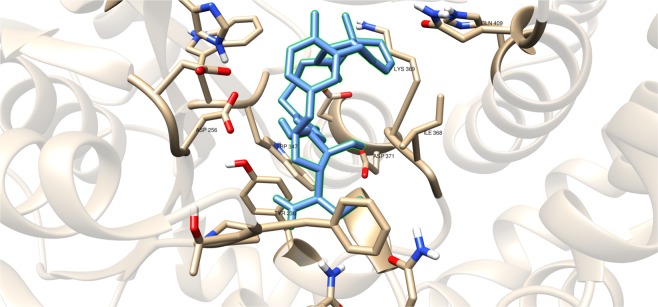
Three-dimensional plot of the interaction of compound #74 with GlfT2’s active site.

Additional amino acids were found to be interacting with these compounds as shown in Tables 18 and 20. It can be seen that compounds #63–65 were found to be interacting with both M286 and K402 through hydrogen bonding (Figs. 18 and 19). These interactions were also observed between compound #59 and K402, and between compounds #68 and #75 and M286 (Figs. 18 and 19).

**Table 20:** Interacting amino acids with  3D-QSAR designed compounds.

Compounds	Major interacting amino acids								Additional interacting amino acids					
**Natural substrate**	Y236	D256	W309	W348	K369	D372	W399	Q409						
**66**	Y236		W348	K369	D372			R171	G232	W347		D371		
**67**	Y236	D256	W348	K369	D372			R171	G232	W347	I368			
**68**	Y236	D256	W348	K369	D372		P167	R171	G232	W347	I368		H396	M286
**69**	Y236	D256	W348	K369		Q409			G232	W347	I368	D371		
**70**	Y236	D256	W348	K369	D372					W347	I368	D371		
**71**	Y236	D256	W348	K369				R171	G232	W347	I368	D371		
**72**	Y236	D256	W348	K369	D372				G232	W347		D371		
**73**	Y236	D256		K369	D372		P167		G232	W347	I368	D371		
**74**	Y236		W348	K369		Q409				W347	I368	D371		
**75**	Y236	D256	W348						G232	W347			H396	M286

### Structure-activity relationship representation

Various designs of the possible GlfT2 inhibitors are summarized in Fig. 21 using a Structure-Activity Relationship (SAR) representation. This shows the effect of the different R-groups added to the pharmacophore of the QSAR-based, Donor substrate-based and Acceptor substrate-based compounds on their activity. To design for possible GlfT2 inhibitors, the compound should have at least one sugar moeity (D-Gal*f*) provided that the 5-OH and/or 6-OH position have R-groups that could disrupt the compounds’ hydrogen-bond interaction with D372 (Figure 18). In addition, the presence of a UDP or UDP-like moiety (long alkyl chain or steriodal aglycon) could possibly increase the binding affinity of the compound.

**Figure 20:**
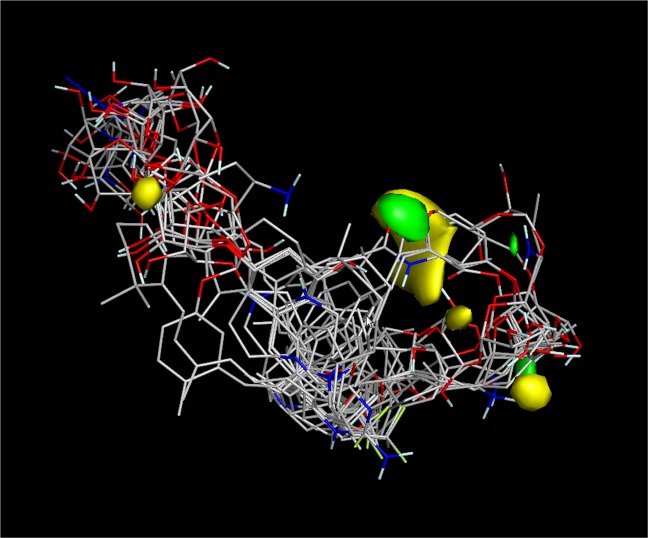
3D-QSAR model for the synthesized GlfT2 compounds (R^2^ = 0.99). Yellow isosurface represents the positive steric field, green isosurface represents the negative steric field.

**Figure 21:**
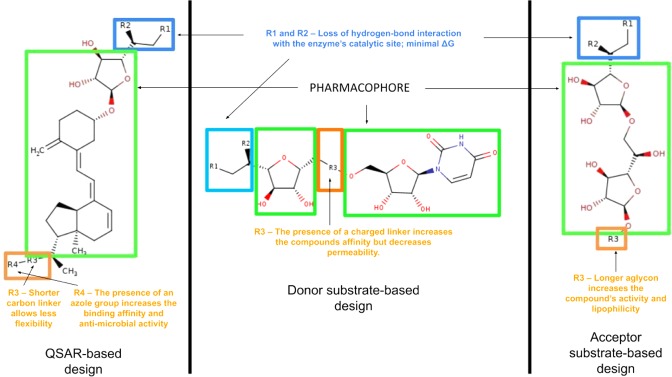
Structure-Activity Relationship (SAR) representation of the QSAR-based design, donor substrate-based design and acceptor substrate-based design compounds.

### ADMETox evaluation of the best candidates

The predicted ADME part of this study was carried out using an online server, SwissADME^67^, that gives values for lipophilicity, water solubility, drug-likeness, medicinal chemistry (i.e. leadlikeness, and PAINS and Breck). Whereas, *in silico* toxicity evaluation was carried out also using an online server, ProTox-II^68^, that gives predicted oral toxicity values, predicted cytotoxicity, mutagenicity, carcinogenicity, hepatotoxicity, and immunotoxicity. In addition, ProTox-II also gives an overview whether the compounds being analyzed will bind to the proteins known to produce adverse reaction to drugs.

#### Drug-likeness, bioavailability, synthetic accessibility and alerts for PAINS and Brenk filters

Drug-likeness is a quantitative parameter that measures a compound’s oral bioavailability. Abbot bioavailability score predicts the chance of a compound to have at least 10% oral bioavailability in rat or measurable Caco-2 cell line permeability experiment. This permeability experiment use Caco-2 cells as a model for human intestinal absorption of drugs^69^. The parameters considered to measure the score are lipophilicity (−0.7 < XLOGP3 < 5.0), molecular weight (MW) (150 g mol^−1^ < MW < 500 g mol^−1^), polarity (20 Å^2^ < TPSA < 130 Å^2^), solubility (0 < log S (ESOL) < 6), saturation (0.25 < Fraction Csp3 < 1) and flexibility (0 < of rotatable bonds < 9). This semi-quantitative rule-based score defines the compounds into four probability score classes i.e 11%, 17%, 55% and 85%^69,70^. The acceptable probability score is 55% which indicates that it passed the rule of five. Among the top hits, compounds #31, #60, #61, #62, #66, #67, #71, #72, #73, #74 and #75 showed a score of 55%, indicating good bioavailability.

PAINS (Pan Assay Interference compounds) and Brenk^71^ method are used to identify potentially problematic molecular fragments that could give false-positive biological activity output^65,69^. Thus, the PAINS and Brenk screening showed that compounds having the following functional groups: (1) imine- and azo- fragments i.e compounds #1, #2, #4, #27, #58, and #59, (2) isolated alkene fragment i.e compounds #31, #58, and #62, (3) thiocarbonyl fragment i.e compounds #3 and #64, and (4) polyene fragment i.e compound #61 (Table 21). The remaining compounds showed no problematic chemical fragments.

**Table 21:** Drug-likeness parameter values for the top hit compounds.

Compound	Drug-likeness						Synthetic Accessibility
	MW (g/mol)	TPSA (Å^2^)	ESOL Log S	Fraction Csp3	#Rotatable bonds	Bioavailability Score	
1	551.63	228.28	−3.55	1	19	0.11	6.9
2	517.61	187.82	−4.57	0.92	18	0.11	6.68
3	584.72	248.67	−2.71	0.96	20	0.17	6.81
4	452.42	269.95	0.89	0.93	12	0.11	5.8
27	635.52	312.43	−3.24	0.22	11	0.17	5.3
31	550.26	97.93	−7.23	0.16	5	0.56	3.92
58	733.96	215.95	−7.45	0.92	14	0.11	8.69
59	731.94	215.95	−6.99	0.82	15	0.11	8.55
60	676.92	138.07	−7.22	0.85	13	0.55	8.61
61	578.73	138.07	−5.28	0.69	12	0.55	7.51
62	688.89	147.3	−7.44	0.69	16	0.55	7.62
63	708.92	178.53	−5.6	0.85	13	0.17	8.72
64	737	208.21	−6.47	0.82	15	0.17	8.42
65	719.7	314.05	−1.87	0.55	17	0.11	6.48
66	730.89	138.07	−7.73	0.85	14	0.55	8.53
67	772.97	138.07	−8.63	0.86	15	0.55	8.96
68	731.88	150.1	−7.1	0.84	14	0.17	8.38
69	543.82	84.94	−7.39	0.82	10	0.17	7.53
70	537.77	84.94	−7.54	0.65	10	0.17	7.07
71	518.69	97.3	−5.91	0.47	10	0.55	6.54
72	533.72	89.87	−6.84	0.48	10	0.55	6.25
73	506.68	97.3	−5.65	0.52	10	0.55	6.74
74	563.73	139.4	−6.24	0.59	10	0.55	6.93
75	539.71	139.4	−5.3	0.7	8	0.55	7.07

Lead-likeness of a compound is predicted using parameters such as MW (250 g mol^−1^ ≤ MW ≤ 350 g mol^−1^), octanol/water partition coefficients (XLOGP ≤ 3.5) and number of rotatable bonds (# rotatable bonds ≤ 7). Results showed that none of the top hit compounds fall within the set criteria. To quantify the complexity of the molecular structure, synthetic accessibility was assessed. The results showed that the scores for the compounds were in the range of 3.92–8.96 (Table 21). The obtained values revealed that the compounds here have complex synthesis route.

#### Absorption, distribution, metabolism and excretion properties evaluation of the top hit compounds

Solubility is one of the major properties influencing absorption. The compound’s aqueous and non-aqueous solubility either is important from the drug development process until oral in-take^67^. Lipophilicity is the effective solubility of a compound into the non-aqueous medium and correlated to various models of drug properties such as adsorption, distribution, metabolism and toxicity^70^. Five available predictive models, i.e iLOGP (implicit log P_*o*/*w*_), XLOGP3 (enhanced atomic/hybrid log P_*o*/*w*_ 3), WLOGP (Wildman and Crippen log P_*o*/*w*_), MLOGP (quantitative-structure log P_*o*/*w*_) and SILICOS-IT were used to evaluate the lipophilicity of the compounds. The mean predicted lipophilicity values from these methods is termed as the consensus log P_*o*/*w*_. A molecule is more soluble if the consensus log P_*o*/*w*_ values is more negative^67^. Results showed that compounds #4, #27, and #65 were soluble in non-aqueous medium (Table 22).

**Table 22:** Predicted absorption parameters in ADME evaluation of top hit compounds.

Compound	Consensus Log P_o/w_	Consensus log S	Solubility class
1	0.89	−3.64	soluble
2	1.99	−4.73	moderately soluble
3	0.41	−2.85	soluble
4	−3.64	0.97	very soluble
27	−1.37	−4.29	moderately soluble
31	5.41	−7.49	poorly soluble
58	3.63	−7.14	poorly soluble
59	3.62	−6.86	poorly soluble
60	5.08	−6.75	poorly soluble
61	3.51	−4.58	moderately soluble
62	5.44	−7.47	poorly soluble
63	3.33	−5.03	moderately soluble
64	3.92	−6.25	poorly soluble
65	−1.63	−2.82	soluble
66	5.57	−7.2	poorly soluble
67	6.26	−8.06	poorly soluble
68	4.74	−6.64	poorly soluble
69	6.2	−7.37	poorly soluble
70	5.91	−7.67	poorly soluble
71	4.3	−6.65	poorly soluble
72	3.63	−6.78	poorly soluble
73	4.22	−5.87	moderately soluble
74	4.06	−6.7	poorly soluble
75	3.58	−5.3	moderately soluble

Some drugs have to be highly water soluble to deliver sufficient amount of the active ingredient. Three models were used by SwissADME to predict water solubility i.e ESOL (Estimated SOLubility), Ali and SILICOS-IT (SwissADME in-house solubility predictor). A qualitative estimation of solubility according to log S scale: <−10 - poorly soluble, <−6 - moderately soluble, <−4 - soluble, <−2 - very soluble, and <0 highly soluble^67^. Based from these predictive models, only compound 65 is predicted to be soluble. Compounds #1, #3, and #4 are predicted to be water soluble while compounds #27, #61, #63, #73 and #75 are predicted to be moderately water soluble. The remaining top hit compounds are predicted to be water insoluble (Table 22).

As the drug is absorbed by the system, it encounters diverse membrane barriers such as hepatocyte membrane, gastrointestinal epithelial cells, blood capillary wall, glomerulus, restrictive organ barriers (e.g. blood-brain-barrier), and the target cell^70^. A molecule is said to be less skin permeant if the value of log K_*p*_ is more negative^67,72^. From the predicted results, compounds #4, #27 and #65 are found to be the least skin permeant (Table 23). Moreover, other parameters used to measure the adsorption and distribution of these drugs is through human intestinal absorption (HIA) or gastrointestinal (GI) adsorption data. These data show that compounds #31, #70, #71, #72, and #73 are predicted to be well-absorbed, whereas, compounds #31, #70, #71, #72, and #73 are predicted as non-brain penetrants (Table 23). None of the top hit compounds was predicted to be blood-brain-barrier (BBB) permeant. This means that compounds being proposed here have a relatively large size and they cannot pass the blood-brain barrier. Also, a compound being non-blood-brain permeant lowers the possibility of causing harmful toxicants in the brain and blood stream when metabolized. The remaining compounds were predicted to be neither absorbed nor penetrated in the brain.

**Table 23:** Predicted distribution parameters in ADME evaluation of top hit compounds.

Compound	GI absorption	BBB permeant	log K_p_ (cm/s)
1	Low	No	−7.93
2	Low	No	−6.4
3	Low	No	−9.23
4	Low	No	−12.16
27	Low	No	−10.31
31	High	No	−5.17
58	Low	No	−6.29
59	Low	No	−6.7
60	Low	No	−5.88
61	Low	No	−6.85
62	Low	No	−5.66
63	Low	No	−8.12
64	Low	No	−7.35
65	Low	No	−12.43
66	Low	No	−5.93
67	Low	No	−5.39
68	Low	No	−6.66
69	Low	No	−4.16
70	High	No	−4.05
71	High	No	−5.76
72	High	No	−4.97
73	High	No	−5.77
74	Low	No	−5.94
75	Low	No	−6.72

After being distributed to the organism’s system, metabolism of these drugs takes place and eventually exit the excreta safely. Metabolism plays an important role in the bioavailability of drugs as well as drug-drug interactions. It is also important to have a better understanding if a certain compound is a substrate or non-substrate of the permeability glycoprotein (P-gp). This protein belongs to the ATP-binding cassette transporters which is important in assessing active efflux through biological membranes. It is also essential to have knowledge of the interaction of molecules with cytochrome P450 (CYP) enzymes as they are involved in drug elimination through metabolic transformation^73^. It has been suggested that CYP and P-gp can process small molecules synergistically to enhance the protection of tissues and organisms^74^. Inhibition of these isoenzymes may result in pharmacokinetics-related drug-drug interactions that could lead to unwanted adverse side-effects by lowering the solubility and the accumulation of the drug or its metabolites. To better understand the mechanism of drug deposition, efficacy and toxicity, the top hit compounds were evaluated to determine whether the compound can act as substrate or an inhibitor of P-gp and CYPs. All compounds are found to be substrates of P-gp except for compounds #1, #2, #4, #61 and #69. Moreover, the top hit compounds presented were found to be substrates of CYP1A2, CYP2C19 and CYP2D6. All compounds are predicted to be CYP2C9 substrates except compounds #31 and #73, whereas, for CYP3A4, compounds #27, #58, #59, #63, #64, #65, #68, and #72 were found to be potential substrates (Table 24).

**Table 24:** Predicted metabolism parameters in ADME evaluation of top hit compounds.

Compound	P-gp substrate	CYP1A2 inhibitor	CYP2C19 inhibitor	CYP2C9 inhibitor	CYP2D6 inhibitor	CYP3A4 inhibitor
1	Yes	No	No	No	No	No
2	Yes	No	No	No	No	No
3	No	No	No	No	No	No
4	Yes	No	No	No	No	No
27	Yes	No	No	No	No	No
31	No	No	No	Yes	No	Yes
58	Yes	No	No	No	No	No
59	Yes	No	No	No	No	No
60	Yes	No	No	No	No	Yes
61	No	No	No	No	No	Yes
62	Yes	No	No	No	No	Yes
63	Yes	No	No	No	No	No
64	Yes	No	No	No	No	No
65	Yes	No	No	No	No	No
66	Yes	No	No	No	No	Yes
67	Yes	No	No	No	No	Yes
68	Yes	No	No	No	No	No
69	No	No	No	No	No	Yes
70	Yes	No	No	No	No	Yes
71	Yes	No	No	No	No	Yes
72	Yes	No	No	No	No	No
73	Yes	No	No	Yes	No	Yes
74	Yes	No	No	No	No	Yes
75	Yes	No	No	No	No	Yes

#### *In silico* toxicity evaluation of top hit compounds

Investigating the ADMET properties of a compound is a critical step for drug development. If a drug passes this step, subsequent toxicity tests are warranted. However, toxicity tests are time consuming and expensive especially if there are significant number of candidate compounds^75,76^. To keep up with increasing demand from the pharmaceutical industries, *in silico* toxicity evaluation is initially used to determine the compound’s toxicity as a fast and an inexpensive method to reduce the number of compounds to be sent later for further testing. *In silico* toxicity evaluation could not act as absolute answer for the compound’s toxicity evaluation^75^. Thus, it should always be accompanied by an *in vitro* and *in vivo* experiments to verify the biological activities beyond the capability of these computational approaches.

Here, the top hit compounds were subjected to an *in silico* toxicity evaluation using Pro-Tox. The LD_50_ is defined as the median lethal dose of a compound at which the test subjects die upon exposure to it. The toxicity class ranges from 1 to 6, 1 being fatal if ingested and 6 being non-toxic^77^. The results showed that the top hit compounds #3, #4, #63, #65, #66, #67, and #73 were predicted to be orally toxic (range between toxicity class 1 to 3) (Table 25).

**Table 25:** Predicted LD_50_ and Toxicity class of the top hit compounds.

Compound	Predicted LD_50_ (mg/kg)	Toxicity class
1	2275	4
2	2275	4
3	50	2
4	250	3
27	1000	4
31	350	4
58	590	4
59	250	3
60	55	4
61	4000	5
62	3000	5
63	55	3
64	5000	5
65	135	3
66	55	3
67	55	3
68	590	4
69	5000	5
70	2500	5
71	2500	5
72	2500	5
73	40	2
74	2500	5
75	590	4

The Pro-Tox online server^68^ also predicts four toxicological endpoints such as cytotoxicity, mutagenicity, carcinogenicity, and immunotoxicity. Results suggested that all the top hit compounds were predicted to be immunotoxic except for compound #31 (Table 26). Immunotoxic chemicals are known to alter the correct functioning of immune system by B cell growth inhibition^68,77^. Moreover, the organ toxicity, specifically hepatotoxicity was predicted to evaluate if the compound will cause liver dysfunction^68,77^. Results showed that the top hit compounds were predicted to be non-hepatotoxic. Moreover, compound #4 was predicted to be a mutagenic compound (Table 26). This means that it can possibly cause alteration of a genetic material, such as the DNA of an organism.

**Table 26:** Predicted activity of the top hit compounds on toxicity endpoints.

Compound	Hepatotoxicity	Carcinogenicity	Immunotoxicity	Mutagenicity	Cytotoxicity
1	Inactive	Inactive	Active	Inactive	Inactive
2	Inactive	Inactive	Active	Inactive	Inactive
3	Inactive	Inactive	Active	Inactive	Inactive
4	Inactive	Inactive	Active	Active	Inactive
27	Inactive	Inactive	Active	Inactive	Inactive
31	Inactive	Inactive	Inactive	Inactive	Inactive
58	Inactive	Inactive	Active	Inactive	Inactive
59	Inactive	Inactive	Active	Inactive	Inactive
60	Inactive	Inactive	Active	Inactive	Inactive
61	Inactive	Inactive	Active	Inactive	Inactive
62	Inactive	Inactive	Active	Inactive	Inactive
63	Inactive	Inactive	Active	Inactive	Inactive
64	Inactive	Inactive	Active	Inactive	Inactive
65	Inactive	Inactive	Active	Inactive	Inactive
66	Inactive	Inactive	Active	Inactive	Inactive
67	Inactive	Inactive	Active	Inactive	Inactive
68	Inactive	Inactive	Active	Inactive	Inactive
69	Inactive	Inactive	Active	Inactive	Inactive
70	Inactive	Inactive	Active	Inactive	Inactive
71	Inactive	Inactive	Active	Inactive	Inactive
72	Inactive	Inactive	Active	Inactive	Inactive
73	Inactive	Inactive	Active	Inactive	Inactive
74	Inactive	Inactive	Active	Inactive	Inactive
75	Inactive	Inactive	Active	Inactive	Inactive

Lastly, toxicity of the compounds depends on the different metabolic mechanisms. Several enzymes could either metabolize the drug therapeutically or lead to the formation of toxic metabolites. Below are the possible targets defined according to Novartis that are linked with adverse drug reactions: Adenosine A2A receptor (AA2AR), Adrenergic beta 2 receptor (ADRB2), Androgen receptor (ANDR), Amine oxidase (AOFA), Dopamine D3 receptor (DRD3), Estrogen receptor 1 (ESR1) and 2 (ESR2), Glucocorticoid receptor (GCR), Histamine H1 receptor (HRH1), Nuclear receptor subfamily 1 group I member 2 (NR1I2), Opioid receptor *κ* (OPRK), Opioid receptor *μ* (OPRM), cAMP-specific 3′, 5′-cyclic phosphodiesterase 4D (PDE4D), Prostaglandin G/H synthase 1 (PGH1), and Progesterone receptor (PRGR)^78^. The results showed that the top hit compounds are non-binders with these protein except for compounds #4 and# 65 which were predicted as binders of Prostaglandin G/H synthase 1 (Table 27).

**Table 27:** Predicted activity of the top hit compounds towards the panel of protein toxicity targets.

Compound	AA2AR	ADRB2	ANDR	AOFA	DRD3	ESR1	ESR2	GCR	HRH1	NR1I2	OPRK	OPRM	PDE4D	PGH1	PRGR
1	Non-binder	Non-binder	Non-binder	Non-binder	Non-binder	Non-binder	Non-binder	Non-binder	Non-binder	Non-binder	Non-binder	Non-binder	Non-binder	Non-binder	Non-binder
2	Non-binder	Non-binder	Non-binder	Non-binder	Non-binder	Non-binder	Non-binder	Non-binder	Non-binder	Non-binder	Non-binder	Non-binder	Non-binder	Non-binder	Non-binder
3	Non-binder	Non-binder	Non-binder	Non-binder	Non-binder	Non-binder	Non-binder	Non-binder	Non-binder	Non-binder	Non-binder	Non-binder	Non-binder	Non-binder	Non-binder
4	Non-binder	Non-binder	Non-binder	Non-binder	Non-binder	Non-binder	Non-binder	Non-binder	Non-binder	Non-binder	Non-binder	Non-binder	Non-binder	Non-binder	Binder
27	Non-binder	Non-binder	Non-binder	Non-binder	Non-binder	Non-binder	Non-binder	Non-binder	Non-binder	Non-binder	Non-binder	Non-binder	Non-binder	Non-binder	Non-binder
31	Non-binder	Non-binder	Non-binder	Non-binder	Non-binder	Non-binder	Non-binder	Non-binder	Non-binder	Non-binder	Non-binder	Non-binder	Non-binder	Non-binder	Non-binder
58	Non-binder	Non-binder	Non-binder	Non-binder	Non-binder	Non-binder	Non-binder	Non-binder	Non-binder	Non-binder	Non-binder	Non-binder	Non-binder	Non-binder	Non-binder
59	Non-binder	Non-binder	Non-binder	Non-binder	Non-binder	Non-binder	Non-binder	Non-binder	Non-binder	Non-binder	Non-binder	Non-binder	Non-binder	Non-binder	Non-binder
60	Non-binder	Non-binder	Non-binder	Non-binder	Non-binder	Non-binder	Non-binder	Non-binder	Non-binder	Non-binder	Non-binder	Non-binder	Non-binder	Non-binder	Non-binder
61	Non-binder	Non-binder	Non-binder	Non-binder	Non-binder	Non-binder	Non-binder	Non-binder	Non-binder	Non-binder	Non-binder	Non-binder	Non-binder	Non-binder	Non-binder
62	Non-binder	Non-binder	Non-binder	Non-binder	Non-binder	Non-binder	Non-binder	Non-binder	Non-binder	Non-binder	Non-binder	Non-binder	Non-binder	Non-binder	Non-binder
63	Non-binder	Non-binder	Non-binder	Non-binder	Non-binder	Non-binder	Non-binder	Non-binder	Non-binder	Non-binder	Non-binder	Non-binder	Non-binder	Non-binder	Non-binder
64	Non-binder	Non-binder	Non-binder	Non-binder	Non-binder	Non-binder	Non-binder	Non-binder	Non-binder	Non-binder	Non-binder	Non-binder	Non-binder	Non-binder	Non-binder
65	Non-binder	Non-binder	Non-binder	Non-binder	Non-binder	Non-binder	Non-binder	Non-binder	Non-binder	Non-binder	Non-binder	Non-binder	Non-binder	Non-binder	Binder
66	Non-binder	Non-binder	Non-binder	Non-binder	Non-binder	Non-binder	Non-binder	Non-binder	Non-binder	Non-binder	Non-binder	Non-binder	Non-binder	Non-binder	Non-binder
67	Non-binder	Non-binder	Non-binder	Non-binder	Non-binder	Non-binder	Non-binder	Non-binder	Non-binder	Non-binder	Non-binder	Non-binder	Non-binder	Non-binder	Non-binder
68	Non-binder	Non-binder	Non-binder	Non-binder	Non-binder	Non-binder	Non-binder	Non-binder	Non-binder	Non-binder	Non-binder	Non-binder	Non-binder	Non-binder	Non-binder
69	Non-binder	Non-binder	Non-binder	Non-binder	Non-binder	Non-binder	Non-binder	Non-binder	Non-binder	Non-binder	Non-binder	Non-binder	Non-binder	Non-binder	Non-binder
70	Non-binder	Non-binder	Non-binder	Non-binder	Non-binder	Non-binder	Non-binder	Non-binder	Non-binder	Non-binder	Non-binder	Non-binder	Non-binder	Non-binder	Non-binder
71	Non-binder	Non-binder	Non-binder	Non-binder	Non-binder	Non-binder	Non-binder	Non-binder	Non-binder	Non-binder	Non-binder	Non-binder	Non-binder	Non-binder	Non-binder
72	Non-binder	Non-binder	Non-binder	Non-binder	Non-binder	Non-binder	Non-binder	Non-binder	Non-binder	Non-binder	Non-binder	Non-binder	Non-binder	Non-binder	Non-binder
73	Non-binder	Non-binder	Non-binder	Non-binder	Non-binder	Non-binder	Non-binder	Non-binder	Non-binder	Non-binder	Non-binder	Non-binder	Non-binder	Non-binder	Non-binder
74	Non-binder	Non-binder	Non-binder	Non-binder	Non-binder	Non-binder	Non-binder	Non-binder	Non-binder	Non-binder	Non-binder	Non-binder	Non-binder	Non-binder	Non-binder
75	Non-binder	Non-binder	Non-binder	Non-binder	Non-binder	Non-binder	Non-binder	Non-binder	Non-binder	Non-binder	Non-binder	Non-binder	Non-binder	Non-binder	Non-binder

## Conclusion

Tuberculosis is still a worldwide health problem due to the emergence of strains of *M. tuberculosis* that are resistant to existing anti-TB drugs. There is now a growing interest in targeting GlfT2, the enzyme responsible for the growth of the galactan chain, an important part of the cell wall. To obtain insights on the different interactions of the synthesized compounds with GlfT2, we did ensemble molecular docking studies and the binding energy values of the synthesized compounds showed a −3.00 kcal to −6.00 kcal mol^−1^ range. Two compounds, #27 and #31, have registered binding energy value of −8.32 ± 0.01 and −8.08 ± 0.01 kcal mol^−1^, respectively. These compounds are synthesized as UGM inhibitors and could possibly inhibit GlfT2. Compounds #1–4 are analogs of a known substrate disaccharide modified at 6-OH and 5-OH position of the non-reducing end. Docking studies showed that these are promising compounds with binding energy values of −10.00 to −19.00 kcal mol^−1^. The synthesized and designed compounds were subjected to 3D-QSAR to improve their structural scaffolds and effective interactions with the GlfT2 active site. Here, 18 newly designed compounds were produced considering all steric and electrostatic descriptors. Furthermore, these 18 compounds were all subjected to molecular docking and showed increased binding energy values from −6.00 to −8.00 kcal mol^−1^. Also, a significant increase on the binding energy value was observed when modifying the aglycon part instead of the sugar moiety. Thus, it is suggested that a modification of the aglycon could a better putative way to design GlfT2 inhibitors.

The drug development process includes ADMETox evaluation to determine if a certain proposed drug can be absorbed or can be toxic, thus, top hit compounds were subjected to *in silico* ADMETox. Compounds #31 and #70–73 are predicted to be well-absorbed and non-blood brain permeant. Moreover, compounds #31 and #73 were considered CYP2C9 inhibitor which could lead to adverse side effects. Compounds #70, #71, and #72 passed the ADME evaluation. Predicted toxicity evaluation showed that only compound #31 was non-toxic and passed all the toxicity endpoints.

## Methods

### Molecular dynamics simulation

Two GlfT2 crystal structures are available in PDB. One is bound with UDP-Gal*f* (PDB ID: 4FIY) and the other one is unbound (PDB ID: 4FIX)^20^. The binding affinity of the natural acceptor substrate, with or without the presence of donor substrate in the active site, is statistically insignificant. Thus, for system simplification, the unbound GlfT2 crystal structure (PDB ID: 4FIX) was used for 100 ns all-atom MD simulation using NAMD software package version 2.10^79^. The protein was parameterized using AMBER ff14SB force field. The system was solvated with TIP3P water model in a box of 15 Å on all sides. Counter ions were added to neutralize the system. The system was simulated in NVT with a temperature of 300 K and with an interval output every 2 fs^80^. Long-range interactions were evaluated using particle mesh Ewald method^81^. Bond constraints were applied using SHAKE algorithm^82^.
